# Patient Navigation and Access to Cancer Care in Guatemala

**DOI:** 10.1200/JGO.18.00027

**Published:** 2018-03-28

**Authors:** David Flood, Anita Chary, Kirsten Austad, Merida Coj, Waleska Lopez, Peter Rohloff

**Affiliations:** **All authors:** Wuqu’ Kawoq, Santiago Sacatepéquez, Sacatepéquez, Guatemala.

## TO THE EDITOR:

In a recent commentary, patient navigation to improve cancer care access in Brazil was discussed.^1^ We wish to share our institution’s experience in Guatemala, where we have used patient navigators since 2009.

Guatemala is a low-/middle-income nation in Central America with a population of 16 million people, approximately half of whom are indigenous Maya. The country has disparities in health care access and outcomes for rural and indigenous groups for numerous reasons, including underfunding of health infrastructure, language and cultural barriers, and economic and political marginalization.

Cancer epidemiology in Guatemala is an emerging field. Although one hospital-based cancer registry has existed for years, recent efforts to develop a population-based cancer registry in Guatemala City for pediatric and adult cancers would make population data available for the first time.^2^ Despite these limitations, in 2013 it was estimated that there were approximately 13,000 incident cases of cancer each year, the most common of which were cancers of the stomach, prostate, and cervix.^3^ For the majority of the adult population without private insurance or social security, the primary cancer referral centers in Guatemala are two large public referral hospitals (Roosevelt and San Juan de Dios) and the private, not-for-profit Instituto de Cancerología (National Cancer Institute [INCAN]), all in Guatemala City. Most elements of care at Roosevelt and San Juan de Dios are free of charge, and the Guatemalan government partially subsidizes cancer care for public-sector patients at INCAN. However, standard medications are sometimes unavailable, radiation therapy infrastructure is insufficient, and direct costs incurred by patients still can be significant.^4^

Most pediatric patients with cancer receive subsidized care at the Unidad Nacional de Oncología Pediátrica (National Pediatric Oncology Unit [UNOP]). UNOP has developed cutting-edge social support models but finds that indigenous children have a higher risk of treatment abandonment.^5^

A fundamental problem of the Guatemalan cancer care system is the weak referral chain. There is little coordination between referral-generating entities and oncology centers, one factor that leads patients to frequently arrive at a treatment center with late-stage disease even when diagnosed earlier through screening programs. Among those patients with cancer who present for care, many do not finish their recommended treatment course.

**Fig 1:**
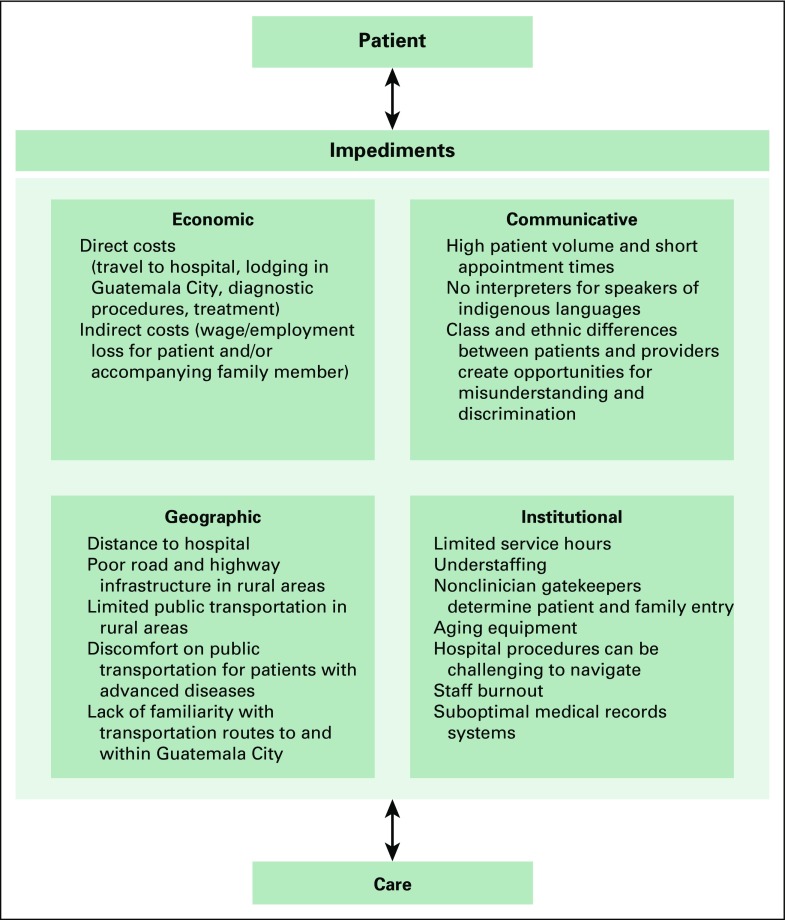
Barriers to engagement and retention in cancer care in Guatemala.

In 2009, our organization developed a patient navigation program to attenuate barriers to cancer care that we had observed among primarily rural and indigenous patients (Fig 1). Patients enter the program from our own primary care clinics and by referral from other nongovernmental clinics and public health centers. This program was initiated specifically as a cancer-care strategy, but, because of positive feedback, we have applied a similar model for other conditions, such as high-risk obstetrics, dialysis, and pediatric cardiovascular disease.^6-8^

Patient navigators serve as the backbone of our cancer referral program. They are involved in all phases of care, including diagnostic evaluation, treatment, post-treatment surveillance, and end-of-life care. Their central goal is to ensure that a patient’s treatment plan is successfully carried out. In practice, patient navigators fulfill the combined roles of interpreter, social worker, case manager, patient advocate, medical records officer, and travel agent. Patient navigators use personal connections, experience, and cultural capital to usher patients through the complex ecosystem of an oncology treatment center. Patient navigators are themselves indigenous and are bilingual in Mayan and Spanish. However, they are not clinically trained and, instead, aim to facilitate the care plan dictated by oncologists and staff physicians.

Since its inception, our patient navigation program has served approximately 300 patients with cancer and 50 patients with high-risk precancerous cervical lesions. Adults represent 80% of the institution’s caseload. On average, our organization spends approximately US$3,000 per case, a sum we raise through general donor fundraising and crowdfunding for each specific patient case. Of note, more than half of our costs are earmarked for nonclinical expenses, such as transportation, lodging, and food. Such expenditures illustrate the need for an integrated approach to addressing barriers to cancer care retention in this setting.

We view our program as an adaptation of patient navigation strategies for vulnerable cancer populations in the United States, where strikingly similar barriers have been described despite the many contextual differences,^9^ as well as an extension of the accompagnateur care model pioneered by organizations like Partners In Health.^10^ Going forward, we plan to improve our program’s data collection, carry out quality-improvement activities, and conduct qualitative research with patients, family caregivers, and health care providers. We are also developing burnout-reduction strategies for patient navigators, who themselves experience significant stress in the course of their daily work.

## References

[1] Bukowski A, Gioia S, Chavarri-Guerra Y Patient navigation to improve access to breast cancer care in Brazil. J Glob Oncol.

[2] Piñeros M, Frech S, Frazier L Advancing reliable data for cancer control in the Central America Four region. J Glob Oncol.

[3] Ferlay J, Soerjomataram I, Ervik M (2013). GLOBOCAN 2012 v1.0, Cancer Incidence and Mortality Worldwide: IARC CancerBase No. 11.

[4] Datta NR, Samiei M, Bodis S (2014). Radiation therapy infrastructure and human resources in low- and middle-income countries: Present status and projections for 2020. Int J Radiat Oncol Biol Phys.

[5] Alvarez E, Seppa M, Rivas S Improvement in treatment abandonment in pediatric patients with cancer in Guatemala. Pediatr Blood Cancer.

[6] Austad K, Chary A, Martinez B (2017). Obstetric care navigation: A new approach to promote respectful maternity care and overcome barriers to safe motherhood. Reprod Health.

[7] Flood DC, Chary AN, Austad K (2017). A patient navigation system to minimize barriers for peritoneal dialysis in rural, low-resource settings: Case study from Guatemala. Kidney Int Rep.

[8] Chary A, Flood D, Austad K Accompanying indigenous Maya patients with complex medical needs: A patient navigation system in rural Guatemala. Healthc (Amst).

[9] Freeman HP, Rodriguez RL (2011). History and principles of patient navigation. Cancer.

[10] Behforouz HL, Farmer PE, Mukherjee JS (2004). From directly observed therapy to accompagnateurs: Enhancing AIDS treatment outcomes in Haiti and in Boston. Clin Infect Dis.

